# Evaluating risk factors for re-exploration due to postoperative neck hematoma after thyroid surgery: a nested case-control study

**DOI:** 10.1007/s00423-019-01836-4

**Published:** 2019-11-18

**Authors:** Farhad Allahyar Salem, A. Bergenfelz, E. Nordenström, J. Dahlberg, O. Hessman, C. I. Lundgren, M. Almquist

**Affiliations:** 1 Department of Clinical Sciences, Lund University, Lund, Sweden; 2 Department of Endocrine Surgery, Sahlgrenska University Hospital, Gothenburg, Sweden; 3 Department of Endocrine Surgery, Uppsala University Hospital, Uppsala, Sweden; 4 Department of Molecular Medicine and Surgery, Karolinska Institute, Stockholm, Sweden

**Keywords:** Thyroid surgery, Postoperative bleeding, Postoperative neck hematoma, risk factors

## Abstract

**Purpose:**

Postoperative bleeding after thyroid surgery remains a potentially lethal complication. Outpatient thyroidectomy is an increasing trend in the high volume centers. There is a need to identify risk factors for postoperative bleeding in order to select proper patients for outpatient thyroidectomy. This study aimed to investigate this issue using a national population-based register.

**Material and method:**

A nested case-control study on patients registered in the Swedish national register for endocrine surgery (SQRTPA) was performed. Patients with postoperative bleeding were matched 1:1 by age and gender to controls. Additional information on cases and controls was obtained from attending surgeons using a questionnaire. Risk factors for postoperative bleeding were evaluated with logistic regression and are presented as odds ratios (ORs) with 95% confidence intervals (CIs). The time of bleeding in relation to surgery was also investigated.

**Results:**

There were 9494 operations, and 174 (1.8%) of them involved postoperative bleeding. In the whole cohort, patients with postoperative bleeding were older, 58 (46–69) vs. 49 (37–62) years, than patients without, *p* < 0.01. Male patients had a higher risk of bleeding, OR 2.18 (95% CI 1.58–2.99). In the case-control cohort, drain was an independent risk factor for bleeding, OR 1.64 (1.05–2.57). Two-thirds of patients bled within 6 h after surgery. The incidence of bleeding after 24 h was 10%.

**Conclusion:**

High age, male gender, and drain are independent risk factors for bleeding after thyroid surgery. Even with careful patient selection, prolonged observation might be necessary in thyroid surgery.

## Introduction

Postoperative bleeding after thyroid surgery is a well-known complication. Despite better knowledge and the ongoing development in surgical techniques, it remains a life-threatening complication. The incidence has been reported as between 0.5 and 4% [1–3]. The percentage of patients requiring immediate re-exploration in order to evacuate hematoma due to respiratory embarrassment is reported to be between 1.2 and 2.1% [4–6]. Re-exploration is associated with morbidity and prolonged hospital stay [7]. In some cases, postoperative bleeding can be superficial and can be managed with a conservative approach and careful observation. However, the clinical presentation of these two patterns of postoperative bleeding cannot always be distinguished [8].

The increasing trend of ambulatory thyroid surgery [9, 10] makes it necessary to study risk factors and predict patients with low and high risks of postoperative bleeding, since this subject remains controversial. The safety of outpatient thyroid surgery is questioned and discouraged by several studies whereas others support such surgery [11–14]. A recent meta-analysis by Khadra H et al. on the safety of same-day thyroidectomy shows that same-day thyroid surgery is safe for certain patients. In their analysis, inpatients were 17.5 times more likely to develop postoperative bleeding than outpatients [13]. Therefore, careful patient selection is necessary when planning for outpatient thyroid surgery [14].

In a retrospective observational study performed recently by Farooq M S et al. on 805 patients who underwent thyroidectomy, the authors concluded that same-day discharge after thyroidectomy should not be recommended as a routine practice because a significant number of bleedings occur after the immediate postoperative period [12].

This study was performed with the aims of identifying risk factors, showing the time pattern of postoperative bleeding, and presenting a subgroup of patients with low risk, suitable for day surgery.

## Material and methods

### Data collection

Data were collected from the Scandinavian Quality Register for Thyroid, Parathyroid, and Adrenal Surgery (SQRTPA), which is the recognized quality register in this medical field in Sweden. The register is endorsed by the Swedish Association of Endocrine Surgeons and the Swedish Association of Otorhinolaryngology and Head and Neck Surgery. Operations performed for thyroid disease registered in the database 2004 to 2010 were included in the study. Data was extracted from the registry in March 2012.

Since not all information needed for the study was available in the SQRTPA, additional information was sought. This meant it was necessary to perform a matched analysis within the cohort, i.e., a nested case-control analysis.

### Definitions

In the SQRPTA, postoperative bleeding is defined as bleeding requiring surgical re-exploration.

### Matching

Cases with postoperative bleeding were matched 1:1 by age and gender to controls. A questionnaire for cases and controls was sent to the attending surgeons in 27 departments. Information was requested regarding patient’s medication (anti-coagulants/anti-platelets); co-morbidities (renal failure, liver dysfunction, and cardiovascular failure); smoking habits; body mass index (BMI); laboratory examinations like (activated partial thromboplastin time (APTT), prothrombin complex international normalized ratio (PK-INR), thrombocytes, and creatinine); the use of a drain; hemostatic technique; the time pattern for postoperative bleeding; and observation of whether bleeding was deep or superficial. In addition, information about further complications after the reoperation was queried. Data from the questionnaires were then merged with the data from the SQRTPA for analysis.

### Analysis

Two types of analysis were performed in order to evaluate the risk factors. First, patients with postoperative bleeding were compared with the cohort in order to assess the impact of age and gender as risk factors. Secondly, patients with postoperative bleeding were analyzed with controls, matched 1:1 as a nested case-control study to study the impact of other risk factors.

After identifying the risk factors, in the whole cohort, patients without risk factors (female gender and age under 50 years) were evaluated regarding frequency of postoperative bleeding. This analysis was not possible for the risk factor (drain) because the information about the use of a drain was only available in the survey data.

The time pattern for postoperative bleeding was classified into four groups: less than 6 h, 6–12 h, 12–24 h, and > 24 h.

Missing values in all the analyses above were included as a separate category, except for the analysis of time pattern for postoperative bleeding in which missing data were excluded.

Other complications after the primary surgery and reoperation were analyzed separately.

### Statistics

For comparison between groups, a Pearson chi-square test was used for categorical variables, and a Wilcoxon rank-sum test for continuous variables. Continuous variables are reported with median and inter quartile range (IQR). Results for comparison within groups are reported as *p* values. Risk factors for patients with postoperative bleeding compared to the rest of the cohort and controls were evaluated using univariable and multivariable logistic regression and are presented as odds ratios (ORs) and 95% confidence intervals (CIs). A *p* value < 0.05 was considered significant. Variables with more than 50% missing values or with too few events were excluded from the multivariable logistic regression model.

For statistical analysis, STATA/IC version 12.1 (StataCorp LP, College Station, TX, USA) was used.

### Ethical issues

The study was approved by the regional ethical committee (DNR 2011/740).

## Results

A total of 9494 operations were registered during the study period. Postoperative bleeding was reported in 174 (1.8%) patients. All patients with bleeding were successfully matched, except for one who could not be matched because of high age (92 years). The response rate for the questionnaire was 96% (332 answers out of 346 sent questionnaires).

### Patients with postoperative bleeding compared with the cohort

Patients with bleeding were older than the rest of the cohort, with a median (Inter Quartile Range (IQR)) age of 58 (46–69) vs. 49 (37–62) years, *p* <0.01. In the univariable logistic regression, high age was a risk factor for bleeding. Patients between 50 and 70 years had an OR of 2.45 with a 95% CI 1.30–4.63 vs. the reference age group, patients < 30 years. Patients over 70 years of age had an OR of 3.50 (1.78–6.87) vs. the reference age group. Male patients had an OR of 2.18 (1.58–2.99) vs female patients. Gland weight between 200 and 500 g had an OR of 2.34 (1.30–4.20) vs. the reference group gland weight < 50 g. Substernal gland had an OR of 1.73 (1.11–2.68) vs. those without. Also, in multiple logistic regression, age over 50 years and male gender remained independent risk factors. Age 50–70 years had an OR of 2.58 (1.33–5.00) vs. the reference age group, patients > 70 years had an OR of 3.62 (1.76–7.43) vs. the reference age group and male gender had an OR of 1.92 (1.38–2.67) vs. female patients, as shown in Table 1.

**Table 1:** Characteristics and risk for bleeding using uni- and multivariable logistic regression in patients operated for thyroid disease 2004–2010 in the Swedish quality register for thyroid surgery (SQRTPA)

Characteristics	Patients with postoperative bleeding *n* = 174(%)	Patients without postoperative bleeding *n* = 9320(%)	Univariable analysis OR (CI)	Multivariable analysis OR (CI)	*P value
Age (years)					
<30	11 (6)	1156 (12)	1.00	1.00	<0.01
30–50	43 (25)	3531 (38)	1.27 (0.65–2.48)	1.38 (0.70–2.71)	
50–70	81 (47)	3463 (37)	2.45 (1.30–4.63)	2.58 (1.33–5.00)	
>70	39 (22)	1170 (13)	3.50 (1.78–6.87)	3.62 (1.76–7.43)	
Gender					
Female	115 (66)	7546 (81)	1.00	1.00	<0.01
Male	59 (34)	1774 (19)	2.18 (1.58–2.99)	1.92 (1.38–2.67)	
Indication for surgery					
Compression symptoms	69 (40)	3605 (39)	1.00	1.00	0.38
Malignancy	27 (16)	1133 (12)	1.24 (0.79–1.95)	1.59 (0.84–3.03)	
Suspect for malignancy	41 (24)	2269 (24)	0.94 (0.63–1.39)	1.05 (0.68–1.62)	
Thyrotoxicosis	37 (21)	2200 (24)	0.87 (0.58–1.31)	1.30 (0.77–2.17)	
Missing	0	113 (1)	na	na	
Type of operation					
Lobectomy	83 (48)	4471 (48)	1.00	1.00	0.68
Thyroidectomy	77 (44)	3910 (42)	1.06 (0.77–1.43)	0.95 (0.62–1.43)	
Thyroid resection	11 (6)	636 (7)	0.93 (0.49–1.75)	0.87 (0.46–1.66)	
Other operations **	3 (2)	303 (3)	0.53 (0.16–1.69)	0.41 (0.11–1.42)	
Operation time (minutes)					
<60	5 (3)	488 (5)	1.00	1.00	0.24
60–120	50 (29)	3055 (33)	1.59 (0.63–4.02)	1.45 (0.57–3.71)	
>120	57 (33)	2919 (31)	1.90 (0.76–4.77)	1.58 (0.60–4.13)	
Missing	62 (35)	2858 (31)	2.11 (0.84–5.29)	1.86 (0.72–4.74)	
Gland weight (grams)					
<50	66 (38)	4149(45.5)	1.00	1.00	0.02
50–200	41 (24)	2230 (24)	1.15 (0.78–1.71)	0.92 (0.59–1.42)	
200–500	14 (8)	378 (4)	2.34 (1.30–4.20)	1.46 (0.75–2.84)	
>500	2 (1)	37 (0.5)	3.39 (0.80–14.39)	2.34 (0.53–10.39)	
Missing	51 (29)	2528(27)	1.26 (0.87–1.83)	1.13 (0.76–1.67)	
Substernal gland					
No	150 (86)	8532 (92)	1.00	1.00	0.01
Yes	24 (14)	788 (8)	1.73 (1.11–2.68)	1.17 (0.71–1.95)	
Sternotomy					
No	172(99)	9275(99.5)	1.00	1.00	0.21
Yes	2 (1)	45 (0.5)	2.39 (0.57–9.95)	1.22 (0.27–5.51)	
Lymph node dissection					
No	147 (84)	7854 (84)	1.00	1.00	0.94
Yes	27 (16)	1466 (16)	0.98 (0.65–1.48)	0.77 (0.44–1.33)	
Thymus operation					
No	173 (99.5)	9276 (99.5)	1.00	1.00	0.84
Yes	1 (0.5)	44(0.5)	1.22 (0.16–8.89)	1.30 (0.17–9.85)	
Previous thyroid operation					
No	157 (90)	8271 (89)	1.00	1.00	0.54
Yes	17 (10)	1049 (11)	0.85 (0.51–1.41)	0.78 (0.44–1.39)	

(*) *P* values present the results of comparison between groups.

(**) Other operations include missing information on operation type and other operation types than lobectomy, resection and total thyroidectomy.

### Patients with postoperative bleeding compared to controls

In the univariable logistic regression, use of a drain was associated with postoperative bleeding, OR of 1.64 (1.05–2.57) vs. patients without. Patients with distant metastasis of thyroid cancer (M1) had an increased risk of bleeding, with an OR of 3.31 (1.16–9.41) vs. patients without (M0). Patients with elevated PK-INR also were at increased risk with an OR of 9.56 (1.13–80.74) vs. patients with normal values. The wide confidence interval was probably due to the fact that there were few observations. The missing values for PK-INR were more than 50% though. In the multivariable logistic regression, only the use of a drain remained significant with an OR of 1.71 (1.07–2.74), as shown in Tables 2 and 4.

**Table 2:** Clinical characteristics and univariable logistic regression of patients with post-operative bleeding and controls using data from (SQRTPA)

Characteristics	Cases *N* = 173 (%)	Controls N = 173 (%)	Odds Ratio (Confidence Interval)	* P value
Indication for surgery				
Compression symptoms	68 (39)	60 (35)	1.00	0.28
Malignancy	27 (16)	32 (19)	0.74 (0.40–1.38)	
Suspect for malignancy	40 (23)	42 (24)	0.84 (0.48–1.46)	
Thyrotoxicosis	38 (22)	35 (20)	0.95 (0.53–1.70)	
Other	0	4 (2)		
Type of operation				
Lobectomy	82 (47)	87 (50)	1.00	0.17
Thyroidectomy	77 (45)	65 (38)	1.25 (0.80–1.96)	
Any type of thyroid resection	11 (6)	14 (8)	0.83 (0.35–1.94)	
Other operations	3 (2)	7 (4)	0.45 (0.11–1.27)	
Operation time (minutes)				
<60	5 (3)	11 (6)	1.00	0.18
60–120	50 (29)	60 (35)	1.83 (0.59–5.62)	
>120	58 (33)	44 (25)	2.85 (0.93–8.95)	
Missing	60 (35)	58 (34)	2.27 (0.70–6.95)	
Gland weight (grams)				
<50	75 (43)	83 (48)	1.00	0.46
50–200	46 (27)	53 (30.5)	0.96 (0.58–1.58)	
200–500	14 (8)	10 (6)	1.54 (0.64–3.69)	
>500	2 (1)	1 (0.5)	2.21 (0.19–24.9)	
Missing	36 (21)	26 (15)	1.53 (0.84–2.77)	
Substernal gland				
No	149 (86)	157 (91)	1.00	0.18
Yes	24 (14)	16 (9)	1.58 (0.80–3.09)	
Sternotomy				
No	171 (99)	173 (100)	1.00	0.15
Yes	2 (1)	0	na	
Lymph node dissection				
No	146 (84)	143 (83)		0.66
yes	27 (16)	30 (17)	0.88 (0.49–1.55)	
Thymus operation				
No	172 (99.5)	172 (99.5)	1.00	1.00
Yes	1 (0.5)	1 (0.5)	1.00 (0.06–16.11)	
Reoperation				
No	156 (90)	148 (86)	1.00	0.19
Yes	17 (10)	25 (14)	0.64 (0.33–1.24)	
Tumour Classification (TNM)	60(100)	62(100)		
pT0	4 (7)	3 (5)	1.00	0.36
pT1	5 (8)	4 (6)	0.93 (0.12–6.87)	
pT2	6 (10)	16 (26)	0.28 (0.04–1.64)	
pT3	14 (23)	13 (21)	0.80 (0.15–4.32)	
pT4	22 (37)	17 (27)	0.97 (0.19–4.93)	
pTx	9 (15)	9 (15)	0.75 (0.12–4.35)	
N0	16 (27)	20 (32)	1.00	0.75
N1	30 (50)	27 (44)	1.38 (0.60–3.21)	
Nx	14 (23)	15 (24)	1.16 (0.43–3.11)	
M0	22 (37)	34 (55)	1.00	0.06
M1	15 (25)	7 (11)	3.31 (1.16–9.41)	
Mx	23 (38)	21 (34)	1.69 (0.38–1.10)	

(*) P-values present the results of comparison between groups.

When analyzing the time pattern of postoperative bleeding, 97 of 152 patients with postoperative bleeding (64%) suffered from bleeding with reoperation within 6 h, and 121 of 152 patients (80%) within 12 h. Fifteen patients (10%) had bleeding with reoperation after 24 h. Information about time pattern was missing for 21 (12%) patients and they were excluded from this analysis, as shown in Table 5.

In the whole cohort, the frequency of postoperative bleeding was 1% if patients with risk factors (male gender and age ≥ 50 years) were removed from the cohort. The frequency for bleeding was the same, 1%, even if patients with the risk factors in the univariable analysis (substernal gland, gland weight 200–500 g and elevated PK-INR) were also removed.

Analysis of other postoperative complications showed no differences between patients with bleeding and controls, as shown in Table 3.

**Table 3:** Information regarding other postoperative complications in patients with postoperative bleeding and controls

Postoperative complications*	Cases *N* = 173 (%)	Controls *N* = 173 (%)	*p* value
Permanent vocal cord palsy			
None	47 (27)	46 (26)	0.13
Unilateral	3 (2)	10 (6)	
Bilateral	1 (0.5)	0	
Missing	122 (70.5)	117 (68)	
Hypoparathyroidism			
No	155 (89)	154 (89)	0.16
Temporary	18 (11)	19 (11)	
Permanent	4 (2)	7 (4)	
Surgical site infection			
Yes	7 (4)	3 (2)	0.19
No	165 (96)	170 (98)	
Missing	1	0	

*Other postoperative complications than bleeding after primary surgery

Although, in total, among patients with bleeding, 35(20%) patients suffered from complications after the re-exploration. These complications were reported as tracheal and teeth injuries, uni- and bilateral vocal cord palsies, surgical site infection, hypoparathyroidism, dysphagia, lung edema, atrial fibrillation, lung embolism, and combination of vocal cord palsy and hypoparathyroidism. One patient was re-reoperated due to re-rebleeding (Tables 4 and 5).

**Table 4:** Univariable and multivariable logistic regression of the patients with postoperative bleeding and controls using survey data

Characteristics	Cases *N* = 173 (%)	Controls *N* = 173 (%)	Univariable analysis OR (95% CI)	Multivariable analysis OR (95% CI)
Body mass index				
Normal (18.5–24.9)	49 (28)	52 (30)	1.00	1.00
Overweight (≥ 25)	58 (33)	62 (36)	0.99 (0.58–1.68)	2.70 (0.39–18.4)
Underweight (≤ 18.4)	4 (2)	2 (1)	2.12 (0.37–12.1)	0.93 (0.53–1.61)
Missing	62 (36)	57 (33)	1.15 (0.67–1.96)	0.93 (0.48–1.80)
Patients’ medications				
Anti-coagulants/anti-platelets				
No	123 (71)	123 (71)	1.00	1.00
Yes	28 (16)	29 (17)	0.96 (0.54–1.71)	0.95 (0.48–1.86)
Missing	22 (13)	21 (12)	1.04 (0.54–2.00)	na
NSAID				
No	143 (83)	149 (86)	1.00	1.00
Yes	7 (4)	4 (2)	1.82 (0.52–6.36)	2.46 (0.60–10.1)
Missing	23 (13)	20 (12)	1.19 (0.63–2.27)	na
SSRI				
No	148 (86)	148 (86)	1.00	1.00
Yes	2 (1)	4 (2)	0.50 (0.09–2.77)	0.47 (0.08–2.73)
Missing	23 (13)	21 (12)	1.09 (0.58–2.06)	na
Cytostatic				
No	149 (86)	150 (87)	1.00	
Yes	0	2 (1)	na	
Missing	24 (14)	21 (12)	1.15 (0.61–2.15)	
Patients’ co-morbidities				
Bleeding disorder				
No	156 (90)	149 (86)	1.00	
Yes	2 (1)	0	na	
Missing	15 (9)	24 (14)	0.59 (0.30–1.18)	
Kidney failure				
No	155 (90)	158 (91)	1.00	1.00
Yes	1 (0.5)	4 (2)	0.25 (0.02–2.30)	0.19 (0.01–2.09)
Missing	17 (10)	11 (7)	1.5 (0.71–1.22)	1.76 (0.06–47.12)
Liver dysfunction				
No	155 (89.5)	162 (94)	1.00	
Yes	1 (0.5)	0	na	
Missing	17 (10)	11 (6)	1.61 (0.73–3.55)	
Cardiovascular disease				
No	142 (82)	150 (87)	1.00	1.00
Yes	14 (8)	12 (7)	1.23 (0.55–2.75)	1.17 (0.44–3.10)
Missing	17 (10)	11 (6)	1.63 (0.74–3.60)	1.66 (0.07–42.27)
Patients’ smoking habits				
None-smoker	66 (38)	72 (42)	1.00	1.00
Smoker	19 (11)	18 (10)	1.15 (0.55–2.38)	1.16 (0.54–2.49)
X-smoker	14 (8)	14 (8)	1.09 (0.48–2.45)	1.06 (0.45–2.51)
Missing	74 (42)	69 (40)	1.16 (0.73–1.86)	1.06 (0.59–1.90)
Laboratory examination (*)				
Elevated PK-INR	7 (4)	1 (0.5)	9.56 (1.13–80.74)	
Missing	124 (72)	116 (67)	1.46 (0.90–2.34)	
Elevated APTT	3 (2)	2 (26)	1.87 (0.29–11.83)	
Missing	134 (77)	126 (73)	1.32 (0.80–2.19)	
Thrombocytopenia	5 (3)	5 (3)	1.07 (0.30–3.82)	0.84 (0.20–3.37)
Missing	72 (42)	65 (38)	1.18 (0.77–1.83)	1.01 (0.61–1.67)
Hemostatic technique				
Ligature	116 (67)	113 (65)	1.00	
Electric instrument	31 (18)	34 (20)	0.88 (0.51–1.54)	0.90 (0.50–1.61)
Ligature and electric instrument	11 (6)	14 (8)	0.76 (0.33–1.75)	0.66 (0.26–1.66)
Missing	15 (9)	12 (7)	1.21 (0.54–2.71)	0.35 (0.05–2.37)
Drain				
No	74 (43)	96 (56)	1.00	
Yes	80 (46)	63 (36)	1.64 (1.05–2.57)	1.71 (1.07–2.74)
Missing	19 (11)	14 (8)	1.76 (0.82–3.74)	1.90 (0.49–7.38)

*A value within the reference zone is the reference for logistic regression

**Table 5:** Time pattern for 152 patients with reoperation for postoperative bleeding

0–6 h	6–12 h	12–24 h	> 24 h
97 patients	24 patients	16 patients	15 patients
64%	16%	10%	10%

## Discussion

This population-based study found that the incidence of re-exploration for postoperative bleeding after surgery for thyroid disease was 1.8%. Age over 50 years, male gender and drain were independent risk factors.

With the increasing trend of outpatient thyroid surgery [9, 10], many other studies have recently been performed to evaluate its safety. A meta-analysis and systematic review on 34 studies concluded that discharging selected patients the same day after thyroid surgery is safe [13].

Weiss A et al. performed, to the best of our knowledge, the largest study in this field in 2014. The study was observational, nationwide, retrospective, and performed on a large population (150,012 patients). The study identified risk factors (older age, male sex, partial thyroidectomy, inflammatory thyroid diseases, chronic kidney disease, bleeding disorders, surgery at a low-volume hospital, black and native American race), useful for predicting patients at risk for postoperative bleeding. The overall mortality rate for patients with bleeding was three times higher than for the entire group of patients [5]. Our study showed additional risk factors that were not found by Weis et al., namely, having a distant metastatic thyroid cancer and receiving a drain. Whether a drain causes the bleeding or is a confounding factor due to the patient selection bias could not be evaluated in this study. Having a drain at surgery could be a marker for a more difficult, “prone to bleeding” operation. Thus, we cannot conclude that drains cause postoperative bleeding.

Our findings regarding drains are similar to the results of a multi-institutional international study performed by Campbell et al. They found that receiving a drain was associated with higher risk of postoperative bleeding. The authors also found that benign thyroid disease, anti-coagulation/anti-platelet medication, use of hemostatic agents, and increased thyroid mass were predictors for postoperative bleeding [15].

Postoperative neck hematoma was not prevented by usage of a drain in neck surgeries other than thyroid surgery [16]. In addition, a drain was associated with more postoperative pain after thyroid surgery in a single-center randomized clinical trial (RCT) performed by Kalemera S E et al. [17].

Rosenbaum M A. et al., in a retrospective observational study performed on 1050 patients who underwent thyroidectomy and/or parathyroidectomy, concluded that in the absence of factors contributing to bleeding, unilateral thyroidectomy could be performed as an ambulatory procedure. The conclusion was based on the extreme low risk of bleeding (0.03%) for patients who underwent unilateral thyroidectomy [18]. Our study does not support this because our data show that the risk of bleeding is almost the same for lobectomy as for total thyroidectomy. This could be because many patients undergo lobectomy when suffering from a unilateral large substernal goiter, which was shown to be a significant risk factor in the univariate analysis in this study.

A previous study on complications after thyroid surgery using data from SQRTPA showed that high age and male gender were independent risk factors for postoperative bleeding [4]. The present study included data from three times more patients and additional information from the attending surgeons, which enabled a nested case-control analysis to be performed and identified another independent risk factor.

Of the patients with postoperative bleeding, 64% were identified within 6 h and 80% within 12 h. Except for the morning procedures, it is difficult to extend observation over 6 h in most settings of day surgery. One-third (36%) of the patients with postoperative bleeding needed re-intervention after 6 h of postoperative care. One-tenth of postoperative hemorrhages were not apparent until more than 24 h after surgery.

Even though the individual risk for late postoperative bleeding is low, some patients will need re-intervention after the end of postoperative observation. Facilities for easy and quick access to professional care for these patients are needed in case of day surgery.

Elevated PK-INR was a significant risk factor in the univariate analysis in this study, although missing data makes this observation unreliable. In clinical practice, it is well known that elevated PK-INR is associated with higher risk for bleeding due to its effect on the coagulation system. Additionally, the abovementioned studies [5, 15] show that the use of anti-coagulants is associated with a higher risk for postoperative bleeding. Therefore, it is not recommended to plan day surgery on patients with elevated PK-INR or those taking anti-coagulants.

Some limitations of the current study are acknowledged. The nested case-control study was retrospective per se, but identification of patients with postoperative bleeding was based on prospective registration of data. There were some missing data for some variables, which may hamper analysis and the interpretation of the results.

Considering the results of this study, female patients under the age of 50, without a substernal goiter, with no drains placed in the wound, gland weight < 200 g, and normal PK-INR before surgery, have a low risk for postoperative bleeding and could be selected for outpatient thyroid surgery. However, the risk for postoperative bleeding is only decreased by 47% in this group, which means that even in this selected group, one out of a hundred patients still needs an intervention for postoperative bleeding.

## Conclusion

Age over 50 years, male gender, and a drain are independent risk factors for postoperative neck hematoma after thyroid surgery and having thyroid cancer with distant metastasis is associated with this complication. Slightly less than two-thirds of the patients bleed within 6 h postoperatively whereas 36% suffer from bleeding beyond the 6-h observation period. Very careful patient selection is mandatory for practicing outpatient thyroid surgery.
